# Life Cycle Assessment of Two Alternative Plastics for Bottle Production

**DOI:** 10.3390/ma14164552

**Published:** 2021-08-13

**Authors:** Patrycja Bałdowska-Witos, Izabela Piasecka, Józef Flizikowski, Andrzej Tomporowski, Adam Idzikowski, Marcin Zawada

**Affiliations:** 1 Department of Technical Systems Engineering, Faculty of Mechanical Engineering, University of Science and Technology, 85-796 Bydgoszcz, Poland; izabela.piasecka@utp.edu.pl (I.P.); fliz@utp.edu.pl (J.F.); a.tomporowski@utp.edu.pl (A.T.); 2 Faculty of Management, Czestochowa University of Technology, 42-200 Czestochowa, Poland; adam.idzikowski@wz.pcz.pl (A.I.); marcin.zawada@wz.pcz.pl (M.Z.)

**Keywords:** life cycle assessment, plastic bottles, environmental impact, quality analysis

## Abstract

The article characterizes selected issues related to the method of performing environmental impact analyses. Particular attention was paid to the need for identifying environmental effects associated with the process of shaping beverage bottles. This study concerns the analysis of selected stages of the machine’s life cycle environmental impact in the specific case of the blow molding machine used in the production of bottles. Life cycle assessment analysis was performed using the SimaPro 8.4.0 software (The Dutch Company Pre Consultants). The CML 2 and ReCiPe2016 methods were chosen to interpret the lists of chemical emissions. Impact categories specific to the CML 2 model are: abiotic depletion, acidification, eutrophication, global warming, ozone layer depletion, human toxicity, fresh water aquatic ecotoxicity, marine aquatic ecotoxicity, terrestrial ecotoxicity, and photochemical oxidation. Among all the considered impact categories, marine aquatic ecotoxicity was characterized by the highest level of potential harmful effects occurring during the bottle production process. A new aspect of the research is to provide updated and more detailed geographic data on Polish bottle production.

## 1. Introduction

Growing public awareness and stricter legal requirements in the field of environmental protection caused an increase in interest in research methods that enable a reduction in the adverse impact on the natural environment and human health of products [1,2]. One such method is the LCA (Life Cycle Assessment) [3].

In light of the urgent need to reduce GHG (greenhouse gas) emissions from food value chains, this document examines GHG emissions from beverage bottle production [4].

The development of knowledge in the field of environmental protection in recent years has shown that the impact of post-consumer packaging on the environment should be considered throughout its life cycle, taking into account many factors that constitute environmental burdens and hazards, among which emissions into the atmosphere of gases containing substances should be distinguished. Others include hazardous conditions, greenhouse gas emissions, water and soil pollution, and excessive use of natural resources. One such method is the Ecological Life Cycle Assessment (LCA). Due to its comprehensive nature, it allows for a full assessment of the environmental impact of the entire life cycle of the selected product from the acquisition of raw materials to the management of waste generated as a result of its use (cradle-to-grave analysis or cradle-to-cradle analysis). According to ISO 14040 and ISO 14044, the LCA test methodology consists of four phases, namely: Goal and Scope Definition, Life Cycle Inventory (LCI), Life Cycle Impact Assessment (LCIA), and Life Cycle Interpretation. These phases are closely related to each other in order to achieve the estimation and ultimately the reduction of the negative environmental impact of a given product or process. One of the traditional areas of application of the LCA is the identification of opportunities to improve the environmental aspects of products at different stages of their life cycle. Considering the above, the subject of this article is the ecological LCA life cycle assessment of two selected PET and PLA bottle units. Packaging, which includes unit, collective and transport packages, is a product “made of any material, intended for storage, protection, transport, delivery or presentation of products, from raw materials to processed goods” [5]. From the ecological point of view, the packaging should have, among others, the following features: minimizing the consumption of raw materials and energy during the production process. They should have a low level of water, soil, and air pollution at the production stage, use phase, and waste management process. They should be as light as possible, as they then take up less space during storage and transport. They should generate as little waste as possible (both in terms of weight and volume) and belong to the applicable organizational and legal system through the use of legible and standardized ecological signs.

Technological progress in the area of polymers from renewable raw materials, as well as consumer expectations for environmental-friendly polymers cause the development of strategic activities aimed at the gradual replacement of the petrochemical raw material on natural raw materials [6,7]. Packaging materials of natural origin, including PLA (polylactide) [8] are produced by plant organisms that absorb CO_2_ from the air, which is needed in the process of photosynthesis with the release of oxygen [9]. Due to the fact that plants absorb CO_2_ from the air during photosynthesis, a zero carbon footprint, or even negative, resulting from the amount of assimilated CO_2_, is assumed for the production stage of plant-derived materials [10]. Starch, after the appropriate chemical modification, has found wide application in the packaging industry. The most commonly used starch is rice and wheat maize. Starch-based thermoplastic materials have found a number of applications in the food packaging industry ranging from extrusion, injection molding, and blow molding. Modified thermoplastic starch is one of the basic materials for creating biodegradable packaging that is constantly undergoing new modifications. Synthetic biodegradable polymers are polylactides, i.e., compounds obtained from corn that are biodegradable through fermentation.

Polylactide has good physical and mechanical properties, which makes it a good candidate for replacing petrochemical thermoplastics [11,12,13]. PET (polyethylene terephthalate) is a polymer material belonging to thermoplastic polyesters with high mechanical strength and high dimensional stability [14]. They are used as packaging material due to a number of important properties of this polymer, especially transparency similar to glass and its low weight, which, combined with flexibility and mechanical resistance, makes packaging made of this material resistant to breakage. An important role in this process is played by the chemical structure of the packaging material, its surface structure, and thickness. The high durability of polymers is an advantage and a disadvantage because the final management hampers its degradation. The degradation mechanism of polymers may be biological, physical, or chemical. Physical degradation occurs through friction or extraction, while chemical degradation occurs through hydrolysis, photolysis, and/or oxidation. Biodegradation is caused by the enzymatic action of bacteria and fungi. The initial stage is polymer degradation during which the chain length is shortened and its fragments are eliminated, and the degree of polymerization and molecular weight are reduced. The result of the process is the production of simple chemical compounds that are nutritious food for microorganisms. The next stage is mineralization. At this stage, microorganisms assimilate the resulting organic compounds, transport them inside the cell, and oxidize carbon to carbon dioxide through metabolic processes. The end products of the biodegradation process of polymers are biomass, i.e., organic material, water, and gases. An important issue about the new packaging materials is not only the way they are produced but also the possibility to close the loop of their life cycle and end-use management that allows recovering the materials for a new production cycle, for example, through the composting procedure. Composting is crucial for packaging polymers as the recycling process requires energy. Composting is a series of processes that use the biodegradability of organic matter to transform it into a specific product, so-called compost. Plastics produced from renewable resources are not necessarily biodegradable, and compostability is positively correlated more with the chemical structure of the compound than with its origin. It is important to distinguish the concept of material biodegradability as being ambiguous with the term compostability. All composting polymers are biodegradable, but the opposite is not the case. The compost generated during the decomposition of plastics must meet certain requirements for fertilizers and must not contain toxic or non-degradable substances.

Authors S. Walker and R. Rothman [15] made a comparative assessment of the life cycle of fossil and biological polymers. Based on the results of the analyses, it was possible to definitely declare differences between the fossil and bio-based polymers. However, it is impossible to definitively declare any type of polymer as having the lowest environmental impact in any of the impact categories. It has been proven that the main sources of variation are the applied methodological procedures of the life cycle assessment and the sources of raw material. In turn, the authors Naomi Horowitz et al. [16] assessed the life cycle of bottled water on four types of bottles: ENSO, PLA, recycled PET, and regular PET (petroleum-based). The results showed that of all fourteen impact categories examined, recycled PET and ENSO bottles were generally better than PLA bottles and regular PET bottles. However, another assessment was carried out by L. Chen [17], who proved that PET bottles based on wood biomass have 21% less global warming potential and require 22% less fossil fuel than their fossil fuel counterparts, but they perform worse in other categories, such as ecotoxicity and ozone depletion. Based on the experience of previous authors, an attempt was made to evaluate the bottle production process only at the forming stage. The study is a comparative study of two types of polymers. Biodegradable polymer under appropriate conditions and 100% PET polymer. The initial assumption is to answer the question whether biodegradable bottles are more environmentally friendly.

The basic research problem is the analysis of the state of knowledge of the issue and collecting and organizing input data of selected phases of the life cycle of the process of shaping bottles for beverages (including materials and media). A new aspect of the research is to provide updated and more detailed geographic data on Polish bottle production.

The work concerns the current issues related to the impact of packaging on the environment. The main goal was to conduct the LCA of beverage bottles made of polyethylene terephthalate and polylactide. The functional unit at the LCA was 1000 bottles for food contact with a capacity of 1000 ml (weight of PET bottles 23.5 kg and PLA bottles 22.8 kg). A limitation of the system has been adopted, wherein the steps of the preforms to provide a production plant for their proper conformation molding of beverage bottles. Further steps of the manufacturing process, such as bottling, labeling, and storage and distribution, were excluded from the system. The LCA analysis was carried out using the program of the Dutch company Pre Consultants called SimaPro 8.4.0. The CML2 and ReCiPe 2016 methods were selected for the interpretation of the lists of released chemical substances. The test results were presented graphically in the form of bar charts and verified and interpreted.

## 2. Materials and Methods

The LCA method is defined as a way of estimating the environmental load from the extraction of raw materials to their end of life. The LCA enables the identification and assessment of emissions of harmful substances as well as the consumption of energy and materials at all stages of the life cycle of the tested object, i.e., in design, production, operation, and decommissioning [6,18].

The main stages are: goal definition, inventory, classification of environmental impacts, and estimation and repair proposals [19,20].

Within the scope of the LCA research, research boundaries, assumptions, and limitations are defined. At this stage of the LCA, it is very important to define the product system that will be the subject of research, product function, and the functional unit [6]. The definition of a product system is the determination of all product-related operations. The unit process is the smallest part of the product system for which data is collected [6].

**Figure 1:**
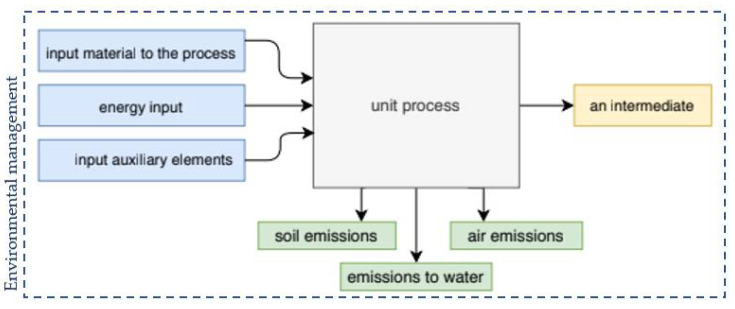
Description of the unit process.

An exemplary description of the unit process is presented in Figure 1.

Its task is to provide a reference for the standardization of system input and output data.

The LCA method consists of four main stages: goal definition, inventory, classification of environmental impacts, and estimation and repair proposals [21,22,23,24,25].

Designing a model to analyze a set of inputs and outputs is the second phase of LCA. The model reflects the entire product system, and its smaller elements represent technological operations. A technological operation should be understood as the smallest part of the system for which information on resources is collected. Data collection enables the precise determination of the source of origin, geographic scope, representativeness, and precision, which are essential elements of the uncertainty analysis.

Taking into account the confidential nature of the LCI results presented in the study and the company’s trade secrets, the values presented in Table 1 were changed by a factor from 0.8 to 1.2. Data on individual stages of the process come from one company in Poland and relate to the bottle shaping process carried out there. The modeling used the Ecoinvent 3.2 database. We have included the input data from a PET bottle in our previous article “Application of LCA Method for Assessment of Environmental Impacts of a Polylactide (PLA) Bottle Shaping”. Doi: 10.3390/polym12020388.

For the adopted system, the exact time period, geographical area, technological type of collected data, and their accuracy and completeness were determined. The included inventory data come from 2017 from the technological line for the production of bottles. The production factory is located in the central part of the country. On this basis, it is possible to determine the precision of the real data accepted for research, which are of high quality.

### 2.1. Characteristics of the Analysis Object

The research object adopted for the analysis is a modern technological line for blowing bottles. The principle of operation of the device is based on bringing the basket filled with the preform to the elevator, which pours the contents of the package into a special dispenser. Then, the preforms are directed to the heating zone by means of a conveyor belt. Heating consists of heating the entire volume of the preform while cooling the area of the necks and the surface at the same time. In the heating module, the air drawn in for cooling the necks is cooled with cold water from the heat exchanger. The heat is distributed evenly throughout the preform. After entering the heating module, the preforms are heated to a temperature of 110 °C until the material becomes plastic. During this time, the preforms are rotated around their axis, which improves the heat distribution inside them. The shielding covers protect the preform thread from overheating. The cooling system for the surface of the preforms and necks covers the entire heating zone and the heating module heating zone. The surface of the preform is cooled with ambient air, which prevents it from overheating. The heating elements heat the preforms with infrared radiation. The electrical connections of the infrared heaters are air-cooled to prevent overheating. The soaking zone in the heating module is used to evenly distribute the heat inside the preform. The heating module installed in the device is equipped with numerous control devices. A pyrometer at the preform transfer point from the heating mandrel chain to the inlet star of the blow module controls the surface temperature of the preforms and threads. Then the preforms are transferred to the blow molding station.

The high-pressure air supplied must be dry, clean, dust-free, and oil-free. The air block is responsible for the proper supply of air to the machine. The stretching and blowing unit controls the air flow via a nozzle during pre- and final blowing of the preforms. In the blow module, the preforms are blown, and as a consequence, effectively shaped bottles are obtained. After the blowing process is finished, the finished beverage bottles leave the machine through the outlet zone.

### 2.2. Determination of Goal and Scope

The most important step in eco-balance technical facilities is to define the goal properly [17]. The purpose of this LCA study is to analyze the environmental loads of selected unit processes occurring in the bottle blowing process based on selected characteristics [6,8]. System boundaries have also been identified that will include subprocesses covering the stages of:

- collecting cold preforms for the furnace;
- heating preforms in the blow molding furnace;
- stretching and lengthening the hot preform;
- cooling and degassing of ready bottles.

The stages of bottle filling, use, collection, storage, and transport have been excluded from the boundaries of the analysis [20].

Data for individual unit processes within the product system limits can be, according to ISO 14040: 2006, included in the following types:

- input data: energy expenditure, raw material expenditure, auxiliary data, and other;
- output data: products, semi-finished products;
- pollutant emissions to air, water, and soil;
- other environmental aspects.

To date, there is no single, universally recognized LCA methodology in the world, and therefore, these methods used in different countries to evaluate the same products sometimes provide different results. In this way, e.g., in the case of eco-balance analyses of packaging materials, it may happen that one test result favors a returnable bottle over another cardboard box, and yet, others define the environmental impact of these products as equal [9,10,11,12].

### 2.3. System Boundary and Functional Unit

The process was divided into six unit operations, taking into account the demand for media and materials. One thousand bottles with a capacity of 1 L were adopted as the functional unit of the study. The scope of analysis included taking preforms into the furnace, heating, stretching, and elongation, as well as pressure preforming, degassing, and cooling of the finished bottle.

**Figure 2:**
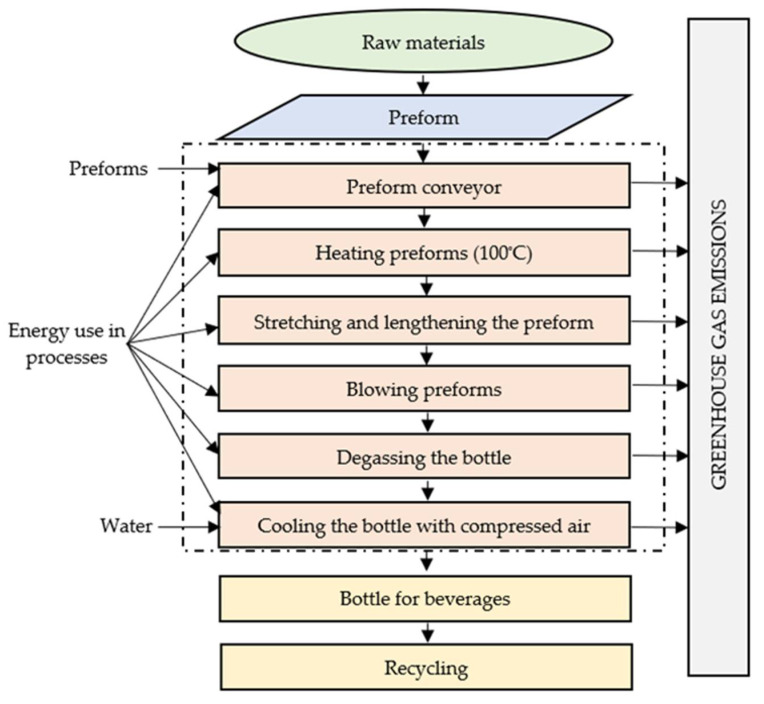
The block diagram of the PET bottle production process.

As a result, technological operations of the adopted processes were charged with the same simplifications, which allowed to take the level of exclusion below 0.01% share in the entire life cycle and share in all potential environmental impacts for each considered technological operation for both types of bottles made of PET and PLA (Figure 2) [3,6,14,15].

### 2.4. Life Cycle Impact Assessment (LCIA)

LCIA is a step for evaluating the potential environmental impacts by converting the LCI results into specific impact indicators. Carrying out the LCIA must follow several sub-steps: The first is the selection of the impact category to be analyzed. The second is the assignment of the LCI results to different impact categories (classification). Third, potential impact indicators (characteristics) are calculated. These three steps are mandatory for the LCIA.

#### 2.4.1. CML 2001 Baseline 2000

**Figure 3:**
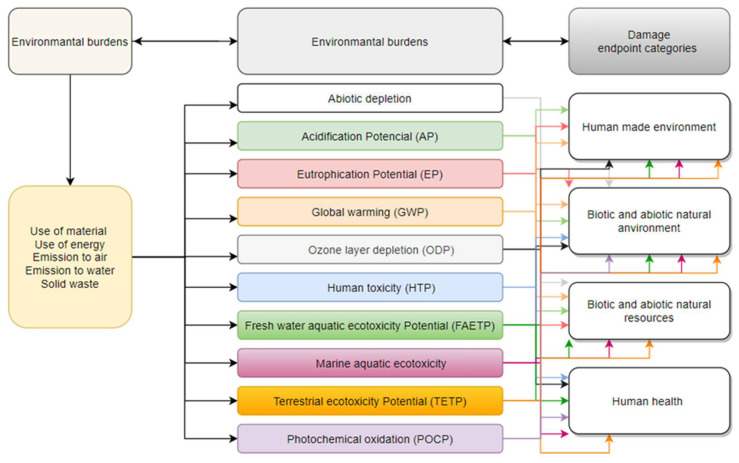
CML 2 baseline 2000 method map.

In the CML 2 method, environmental burdens are aggregated according to the environmental impact they can potentially cause. This method takes into account midpoint categories defined as midpoint categories. We distinguish among them the following categories (Figure 3):

- Abiotic depletion;
- Acidification Potential (AP);
- Eutrophication Potential (EP);
- Global warming (GWP);
- Ozone layer depletion (ODP);
- Human toxicity (HTP);
- Fresh water aquatic ecotoxicity Potential (FAETP);
- Marine aquatic ecotoxicity;
- Terrestrial ecotoxicity Potential (TETP);
- Photochemical oxidation (POCP).

Acidification describes the change in soil acidity caused by the deposition of sulfates, nitrates, and phosphates in the atmosphere [23]. The main acidifying substances are NO_X_, NH_3_, and SO_2_. This includes all relevant substances because there are no emissions of other acidifying substances such as HCl, HF, etc., in the foreground system [6,27,28,29].

(1) $$\mathrm{AP}=\overset{J}{\underset{j=1}{\sum }}AP_{j}B_{j}\;{\mathrm{kg}\;\mathrm{SO}}_{2}\;\mathrm{eq}$$

where $AP_{j}$ represents the acidification potential of gas *j* expressed relative to the AP of SO_2_, and *B_j_* is its emission in kg.

Eutrophication can be defined as the enrichment of nutrients in the aquatic environment [30]. In inland waters, eutrophication is one of the main factors determining its ecological quality.

(2) $$\mathrm{EP}=\overset{J}{\underset{j=1}{\sum }}EP_{j}B_{j}\;{\mathrm{kg}\;\mathrm{PO}}_{4}^{3-}\;\mathrm{eq}$$

where $EP_{j}$ represents their respective eutrophication potentials—$B_{j}$—in a special emission, such as nitrogen (N), nitrogen oxides (NOx), ammonium (${\mathrm{NH}}_{4}^{+})$, phosphate (${\mathrm{PO}}_{4}^{3-}),$ phosphorus (P), and chemical oxygen demand (COD). EP is expression relative to ${\mathrm{PO}}_{4}^{3-}$.

Another term in the literature is “summer smog” [14]. The photooxidants produced in the process are from the photochemical team that affects health and ecosystems [10]. This ground-level ozone is created in the atmosphere by nitrogen oxides and volatile cards in the presence of sunlight [30,31,32].

The values of the GWP of the time depend on horizon over which the global warming effect is assessed. GWP factors for shorter times (20 and 50 years) provide an indication of the short-term effect of greenhouse gases on the climate, while GWP for longer periods (100 and 500 years) are used to predict the cumulative effects of these gases on the global climate [33].

(3) $$\mathrm{GWP}=\overset{J}{\underset{j=1}{\sum }}GWP_{j}B_{j}\;{\mathrm{kg}\;\mathrm{CO}}_{2}\;\mathrm{eq}$$

where $B_{j}$ represents the emission of greenhouse gas. $GWP_{j}$ represents the factors for different greenhouse gases that are expressed relative to the global warming potential of CO_2_, which is, therefore, unity.

Human toxicity potential is calculated by taking into account releases toxic to humans in three different media: water, air, and soil:

(4) $$\mathrm{HTP}=\overset{J}{\underset{j=1}{\sum }}HTP_{jA}B_{jA}+\overset{J}{\underset{j=1}{\sum }}HTP_{jW}B_{jW}+\overset{J}{\underset{j=1}{\sum }}HTP_{jS}B_{jS}\;\mathrm{kg}\;1,4-\mathrm{DB}\;\mathrm{eq}$$

where $HTP_{jA},\;HTP_{jW}$, and $HTP_{jS}$ are toxicological classification factors for substances emitted to air, water, and soil, respectively, and $B_{jA},\;B_{jW},\;\mathrm{and}\;B_{jS}$ represent the respective emissions of different toxic substences into the three environmental media [32,33]. The reference substance for this impact category is 1,4-dichlorobenzene.

Photochemical oxidants creation potential is related to the potential of valolite organic compounds and NO_x_ to generate photochemical or summer smog. It is usually expressed relative to the POCP classification factors of ethylene and can be calculated as:

(5) $$\mathrm{POCP}=\overset{J}{\underset{j=1}{\sum }}POCP_{j}B_{j}\;\mathrm{kg}\;\mathrm{ethylene}\;\mathrm{eq}$$

where $B_{j}$ is the emission of species *j* participating in the formation of summer smog, and *POCP_j_* is its classification factor for photochemical oxidation formation [6,15].

Abiotic resources depletion potential includes the depletion of fossil fuels, metlas, and minerals. The total impact is calculated as:

(6) $$\mathrm{ADP}=\overset{J}{\underset{j=1}{\sum }}ADP_{j}B_{j}\;\mathrm{kg}\;\mathrm{SB}\;\mathrm{eq}$$

where $B_{j}$ is the quantity of abiotic resources *j* used, and *ADP_j_* represents the abiotic depletion potential of that resource. This impact category is expressed in kg of antimony used, which is taken as the reference substance for this impact category [34]. Alternatively, kg oil eq can be used instead.

The potential of emissions of chlorofluorohydrocarbons (CFCs) and other halogenated hydrocarbons to deplete the ozone layer is expressed as:

(7) $$\mathrm{ODP}=\overset{J}{\underset{j=1}{\sum }}ODP_{j}B_{j}\;\mathrm{kg}\;\mathrm{CFC}-11\;\mathrm{eq}$$

where $B_{j}$ is the emission of ozone depleting gas *j*. The ODP factors are expressed relative to the ozone depletion potential of CFC-11.

The ecotoxicity to fresh waters indicator refers to the impact on fresh water ecosystems as a result of the emission of toxic substances to air, water, and soil [6,35]. Ecotoxicity potential (FAETP) is calculated using USES-LCA, describing the fate, exposure, and effects of toxic substances. The time horizon is infinite [29]. Characterizing factors are expressed as 1,4-dichlorobenzene equivalents/kg emissions. The indicator is applicable on a global/continental/regional and local scale [10,14].

#### 2.4.2. ReCiPe 2016

##### Fine Particulate Matter Formation

The selected impact assessment methodology that was applied in SimaPro 8.4.1. software is the ReCiPe midpoint (H). Out of set of 18 midpoint impact categories, one is discussed in this paper as it represents both GHG emissions and air quality in urban environments. This particulate matter formation highlights the impact of primarily formed particulates as well as particulates formed by the condensation of nitrogen oxides, sulfur oxides, ammonia, and non-methane volatile organic compounds (secondary PM). PMF is represented by the emission of PM10-equivalents, i.e., particles, with an aerodynamic diameter smaller or equal to 10 micrometers—the presence of such particles in the air increases the probability of the occurrence of respiratory diseases.

Fine solid particles with a diameter smaller than 2.5 μm (PM2.5) are a complex mixture of organic and inorganic substances. Studies (Particulate studies) show that the mortality effects of chronic PM exposure probably result from <PM2.5 than from coarser particles. Particles with a diameter of 2.5–10 μm (PM2.5–10) are associated with respiratory diseases [26,35,36].

##### Characterization Factors at Midpoint Level

In the middle points characterizing the factors harmful to human health caused by PM2.5, it is important to accept the pollution because the impact and damage are independent of the precursor substance. The inlet fraction (iF) of fine particulates due to emissions in the region and is determined as precursor *x* (iF*x*,*i*) [8]. Particulate formation potentials (*PMFP*) are expressed in primary PM2.5 equivalents by dividing the iFx and the emission weighted global average iF PM2.5:

(8) $$PMFP_{x,i}=\frac{{\mathrm{iF}}_{x,i}}{{\mathrm{iF}}_{\mathrm{PM}\;2.5,\;\mathrm{world}}}$$

### 2.5. Interpretation

The interpretation of the results of the analysis consisted in the mutual assessment of results and loads. The input data necessary for the interpretation used for the analysis were complete and came from a company producing bottles for beverages [14]. During the analysis, the shares of factors of significant importance at individual stages of the bottle shaping process were determined. In addition, the completeness of the LCI data was checked by analyzing mass and energy balance equations [35]. On this basis, compliance with the purpose and scope of work and correctness of the LCA methodology was used. The final analysis results obtained are presented in Section 3.1.

## 3. Results

The results of analyzes carried out as part of Life Cycle Impact Assessment (LCIA) are compiled into two sections including CML 2 and ReCiPe 2016 (Section 3.1).

### 3.1. Characterization Analysis Results

Table 2 presents the total share of the analyzed bottles at the production stage in relation to the functional unit adopted in the work. Based on the obtained results, it is proved that the marine aquatic ecotoxicity category, which is characterized by the highest level of environmental loads, is of key importance for the natural environment in the entire process of shaping the correct packaging. Despite the many advantages of the PET bottles that, e.g., have the best barrier to gases but low water vapor, the biggest problem is that it usually becomes waste as soon as the consumer uses the product. Doing so destroys the concept of a circular economy. Leaving a plastic bottle in the environment means that the decomposition process will take from 100 to even 1000 years, depending on the material and form of the bottle. Hence, it becomes crucial to recognize the problem of the production of plastic bottles, the level of environmental harmfulness of which is 10 times higher than that of bottles made of biodegradable plastics under certain conditions.

As part of the research using CML 2, a detailed analysis of ten impact categories, characteristic of this model, was carried out: abiotic depletion, acidification potential, eutrophication potential, global warming, ozone layer depletion, human toxicity, fresh water aquatic ecotoxicity potential, marine aquatic ecotoxicity, terrestrial ecotoxicity potential, and photochemical oxidation.

The results were compiled for the bottle production process, which included six technological operations: preform before heating, heating the preform, stretching and extension of the preform, pressure shaping of the preforms, degasification of the bottle, and bottle cooling during the production of PET and PLA bottles.

The first step of the analysis included assessing which of the ten categories considered could potentially be the source of the largest number of negative (or positive) environmental consequences in the bottle shaping process.

Among the factors that may negatively affect the quality of the ecosystem, the highest level of harmful impacts was characterized by a group of metal compounds that could lead to toxic environmental effects.

During the production of bottles, significant gaseous emission gets into the environment, including carbon dioxide and sulfur dioxide.

In the group of factors affecting the reduction of environmental quality, fine particulate matter formation is of key importance, where the primary source of PM2.5 fine dust emissions are sources belonging to the cooling of the bottle with compressed air operation, in which the largest part of the emissions is related to the cooling of the finished PET bottle.

When starting the analyses under impact categories, special attention was paid to environmental consequences in the bottle production cycle. It was noticed that one impact category, marine aquatic ecotoxicity (total 456,003.83 kg 1,4-DB eq for PLA bottle and total 481,609.65 kg 1,4-DB eq for PET bottle), was characterized by potentially the highest level of potential harmful effects on the environment. This is the result of a high energy demand in the production processes of disposable bottles and the directly related, highly energy-consuming processes of extracting non-renewable raw materials necessary in individual processes during the production of these packaging.

**Figure 4:**
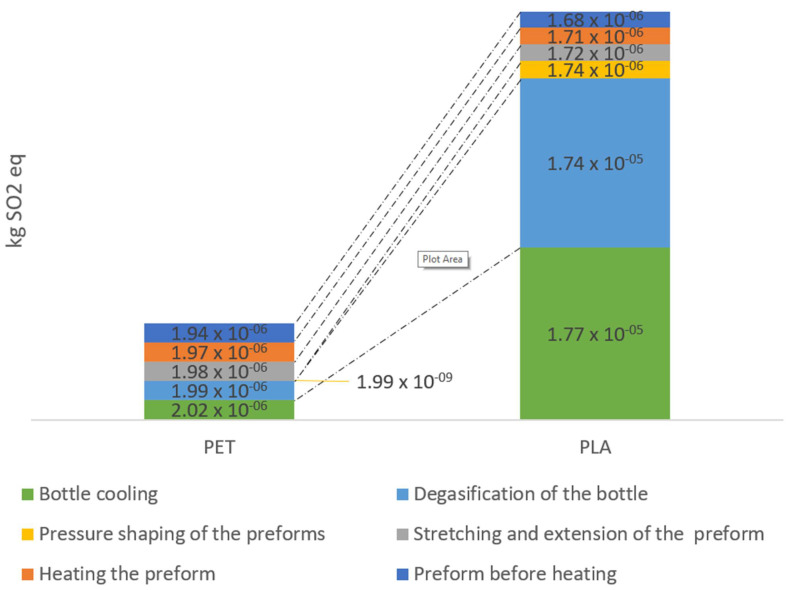
Acidification emissions throughout the entire bottle shaping process.

Analyzing category acidification for each technological operation, lower negative environmental impacts were observed throughout the entire cycle of the PLA bottle shaping process (Figure 4). For performing before heat operation, the effect value was 1.68 × 10^−6^ kg SO_x_/1000 bottles for the PLA preform and 1.94 × 10^−6^ kg SO_x_/1000 bottles for the PET preform. The total amount of negative environmental impacts associated with the emission of sulfur oxides into the atmosphere in the process of shaping beverage bottles was 1.77 × 10^−5^ kg SO_x_/1000 bottles for PLA preform and 2.02 × 10^−6^ kg SO_x_/1000 bottles for PET preform.

**Figure 5:**
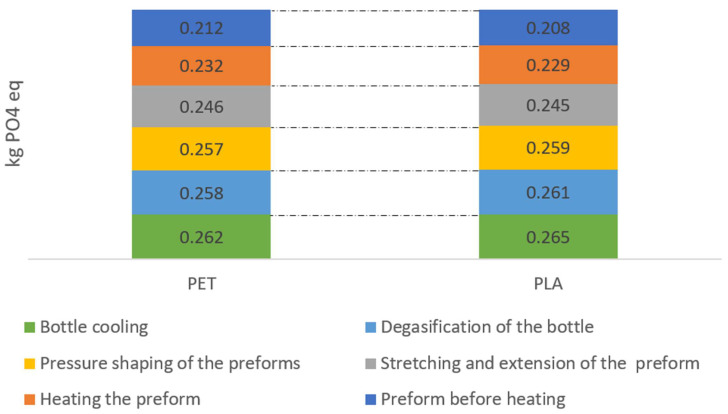
Eutrophication emissions throughout the entire bottle shaping process.

Analyzing the category of eutrophication for each technological operation, similar values of environmental influences were observed throughout the whole life cycle (Figure 5). The lowest calcium phosphorus emission value for the preform conveyor PLA process was 0.208 kg PO_4_/1000 bottles and 0.212 kg PO_4_/1000 bottles of PET. Higher levels of calcium phosphorus emissions were recorded for PLA bottle shaping operations, i.e., stretching and lengthening the preform 0.245 kg PO_4_/1000 bottles, blowing preforms 0.259 kg PO_4_/1000 bottles, degassing the bottle 0.261 kg PO_4/_1000 bottles, and cooling the bottle with compressed air 0.265 kg PO_4_/1000 bottles.

**Figure 6:**
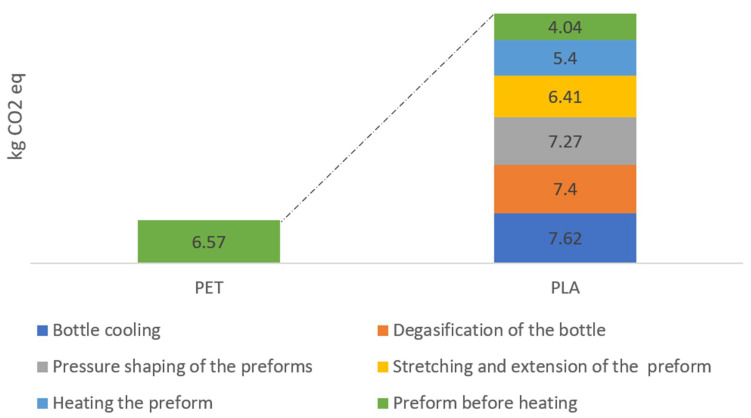
Global warming emissions throughout the entire bottle shaping process.

The share of biodegradable plastic in the bottle shaping process has a positive effect on the size of gas emission indicators. The value of the greenhouse gas emission index preform before the heating process is about 6.57 g CO_2_/1000 bottles for all PET bottle, while with PLA material, this value is reduced to about 4.04 g CO_2_/1000 bottles (Figure 6). The total CO_2_ emission from PLA production is higher than PET. The results show that both energy consumption and CO_2_ emissions would increase significantly if plastic products were to be replaced by those made of biodegradable materials under appropriate conditions. The examples of bottles made of PLA and their comparison with PET bottles show a large impact of bottle forming conditions and overall life cycle GHG performance. With current waste management solutions, PET bottles contribute less to global warming than bottles made of PLA. If plastic bottles are not disposed of in landfills, the results may be quite different. Depending on the waste management method used, the difference between the minimum and maximum CO_2_ equivalent emissions can be significant.

**Figure 7:**
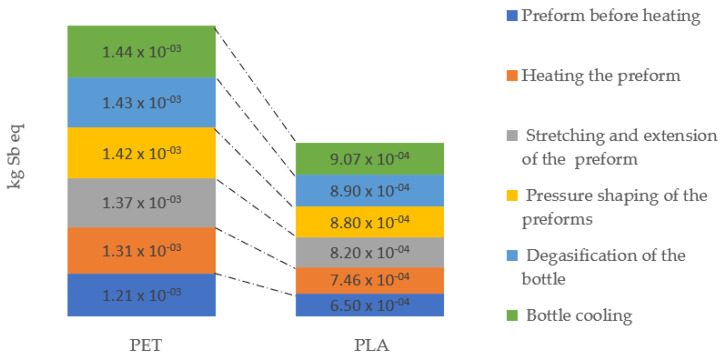
Abiotic depletion emissions throughout the entire bottle shaping process.

Abiotic resources are natural resources (including energy resources), such as iron ore and oil, which are considered inanimate. The depletion of abiotic resources includes both the use of non-renewable and renewable abiotic agents (wind, running water, etc.). This study focuses on the depletion of abiotic resources as defined in the classical LCA methodology, where only non-renewable sources are considered. Operations describing the process of making PLA bottles are distinguished by the lowest level of negative influence under this impact category. The process of producing a bottle made of polyethylene terephthalate, for which the highest negative levels of interaction were observed for all six operations, is of key importance during the shaping of these quantities (Figure 7).

**Figure 8:**
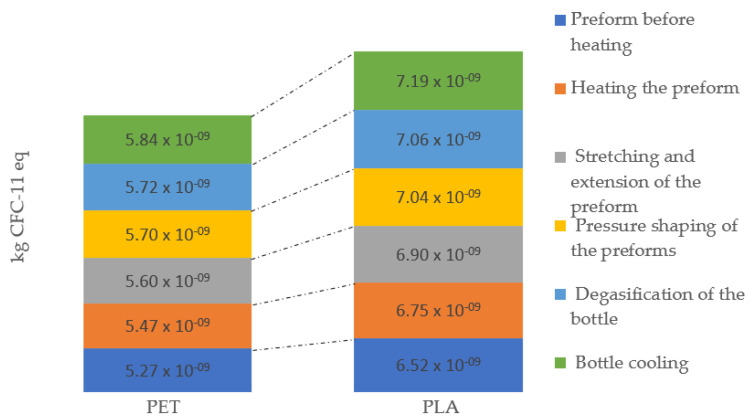
Ozone layer depletion emissions throughout the entire bottle shaping process.

Anthropogenic emissions can deplete stratospheric the ozone. Scientists have carried out systematic observations since the early 1970s, showing that the thickness of the ozone layer is being reduced. Its progressive degradation may have serious consequences on the environment. One of the results of such action is, for example, greater heating of the Earth’s surface and increased impact on human health, increasing the incidence of skin cancer and eye diseases. Another aspect of environmental deterioration is the reduction in the amount of plankton in the seas and oceans, which is the main food for animals living in the seas and oceans. The highest level of emissions of compounds causing the enlargement of the ozone hole was recorded in the process of shaping the PLA bottle at the stage of cooling the finished product (7.19 × 10^−9^ kg CFC-11 eq), while the lowest level of potential impacts was recorded at the stage preform before heating (5.27 × 10^−9^ kg CFC-11 eq) for the PET bottle (Figure 8). Their potentially negative impact causes ozone depletion of the environment. Another equally important aspect is their impact on human health. They can cause diseases of the nervous system and human internal organs. In some cases, they are still used as solvents for specific chemical reactions, and to prevent this, it is recommended to discontinue their use.

**Figure 9:**
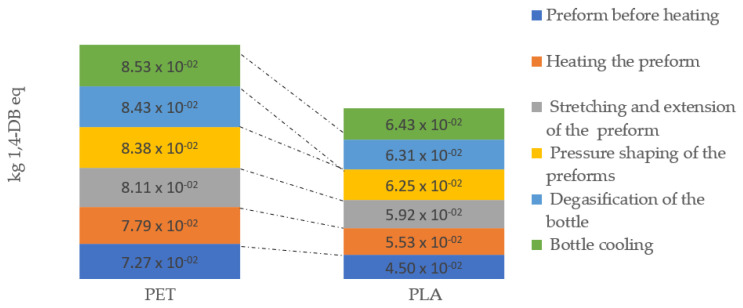
Human toxicity emissions throughout the entire bottle shaping process.

Ecotoxicity is a characteristic of substances that have properties, such as reactivity, with the difference that they require a longer time to develop, and the effects of their polluting effects on the environment last longer. These substances can contribute to the deterioration of the quality of the environment, which poses a real threat to humans. Of all the types of waste generated during the production of beverage bottles, most ecotoxic emissions were recorded for operation bottle cooling in the PET bottle formation process (8.53 × 10^−2^ kg 1,4-DB eq) and the least for preform before heating operations during bottle production PLA (4.50 × 10^−2^ kg 1,4-DB eq) (Figure 9).

As a result of the conducted experiment, the size of the total environmental impact was mainly determined by cadmium emissions has been proven. Cadmium is one of the most important environmental pollutants. Toxic activity is demonstrated by free cadmium ions. Cadmium is usually accumulated in the kidneys and liver. It is especially dangerous due to its rapid absorption by living organisms and easy accumulation in the tissues of plants and animals. Cadmium also contributes to changes in the functionality of cell membranes, inhibits cell division, and reduces the efficiency of photosynthesis.

**Figure 10:**
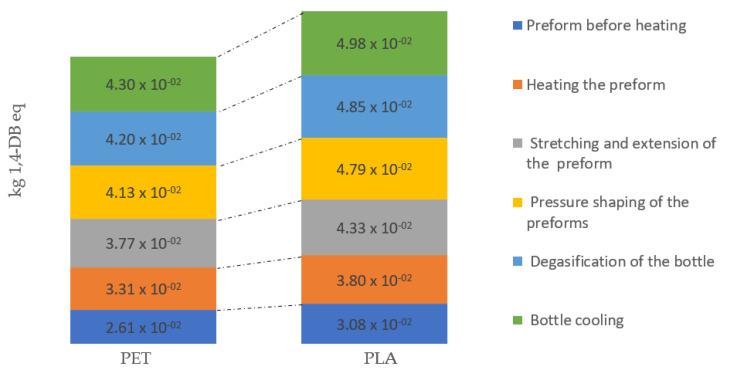
Freshwater aquatic ecotoxicity emissions throughout the entire bottle shaping process.

Freshwater ecotoxicity showed the highest potential emission value determined for the bottle cooling, where the bottle cooling process of PET generated 20% more emission compared to PLA (Figure 10).

**Figure 11:**
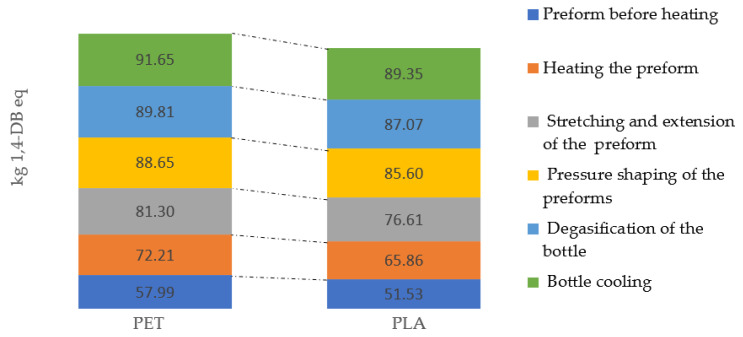
Marine aquatic ecotoxicity emissions throughout the entire bottle shaping process.

Marine ecotoxicity has potentially the highest negative impact on the aquatic environment. The highest similar emission levels were recorded for the degasification of the bottle, which was 91.65 kg 1,4-DB eq in PET bottle and 89.35 kg 1,4-DB eq PLA bottle operations (Figure 11).

**Figure 12:**
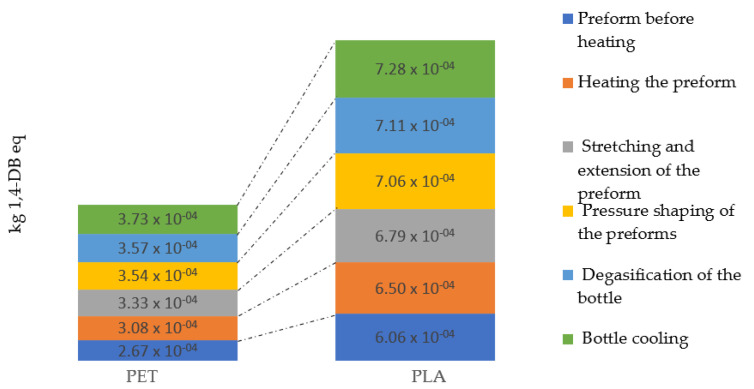
Terrestrial ecotoxicity emissions throughout the entire bottle shaping process.

The occurrence of terrestrial ecotoxicity is caused by lowering of the pH value. This phenomenon occurs as a result of the ecological imbalance in the processes of energy and matter exchange between elements of ecosystems. The reason for these changes is the presence of chemicals. Regular ground acidification occurs when gases such as CO_2_ or SO_2_ are absorbed by the water and react to form acidic compounds at the surface of the earth. The magnitude of the impact of the process of shaping bottles from biodegradable material (7.28 × 10^−4^ kg 1,4-DB eq) was greater than in the case of shaping PET bottles (3.73 × 10^−4^ kg 1,4-DB eq) (Figure 12).

**Figure 13:**
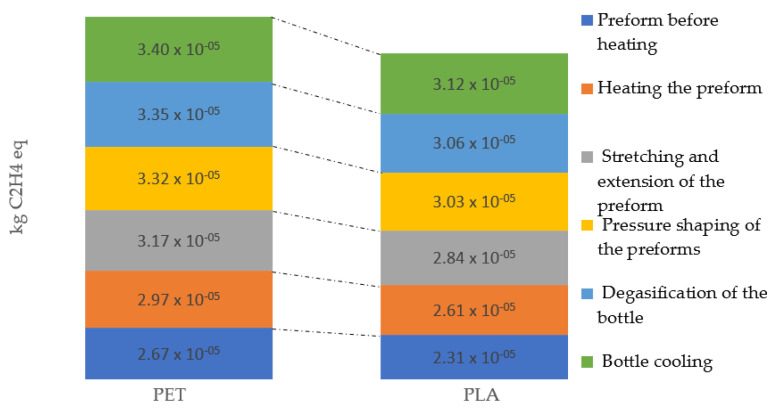
Photochemical oxidation emissions throughout the entire bottle shaping process.

In the case of impact category, photochemical oxidation, similar values of potential environmental impacts were observed. However, the greatest impact on the reduction of environmental quality caused by photochemical oxidation is characteristic for the cycle of PET bottle formation (3.40 × 10^−5^ kg C_2_H_4_ eq). The process of creating a PLA bottle (3.12 × 10^−5^ kg C_2_H_4_ eq) stands out with the lowest level of negative influences under this impact category (Figure 13).

**Figure 14:**
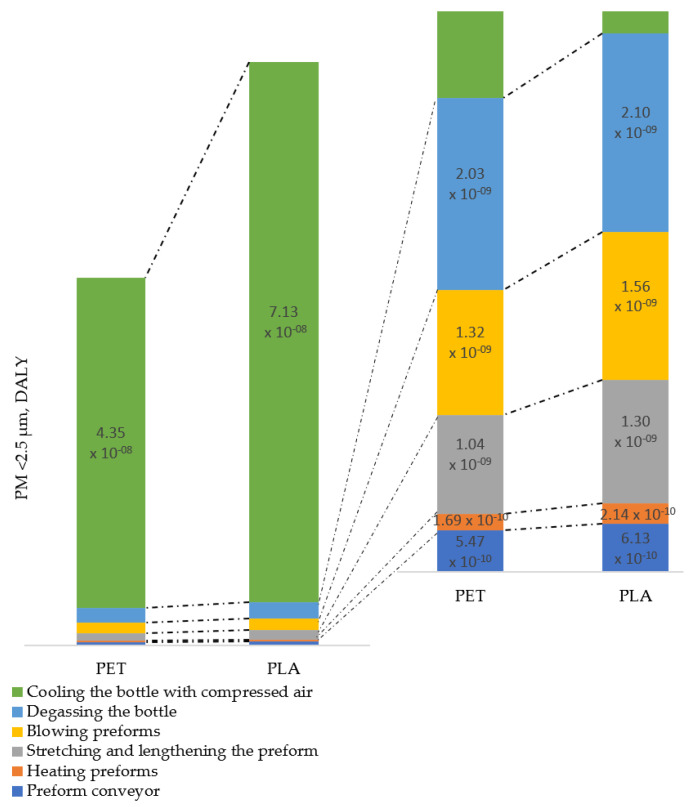
PM < 2.5 µm dust emissions to air and its impact on human health.

Human health and quality of life are closely related to the state of the environment. On the one hand, a good quality environment has a significant impact on both the physical and mental condition of man and on the state of social well-being, while on the other, its degradation and pollution can have a significant negative impact on health. DALY health condition is calculated as the sum of the Years of Life Lost (YLL) due to premature mortality in the population and the Years Lost due to Disability (YLD). Considering all life cycle operations of the PET and PLA bottle shaping process and its impact on human health, it should be stated that the highest potential impact was recorded for the cooling the bottle with compressed air PLA bottle operation at 7.13 × 10^−8^ DALY and PET bottle at 4.35 × 10^−8^ DALY (Figure 14). The reason for this is the share of tap water at the stage of cooling the finished bottle and the amount of electricity consumed. Lower PM emission levels <2.5 µm with similar potential impacts on human health were noted for the following processes: degassing the bottle, blowing preforms, stretching and lengthening the preform, heating preforms, and preform conveyor.

**Figure 15:**
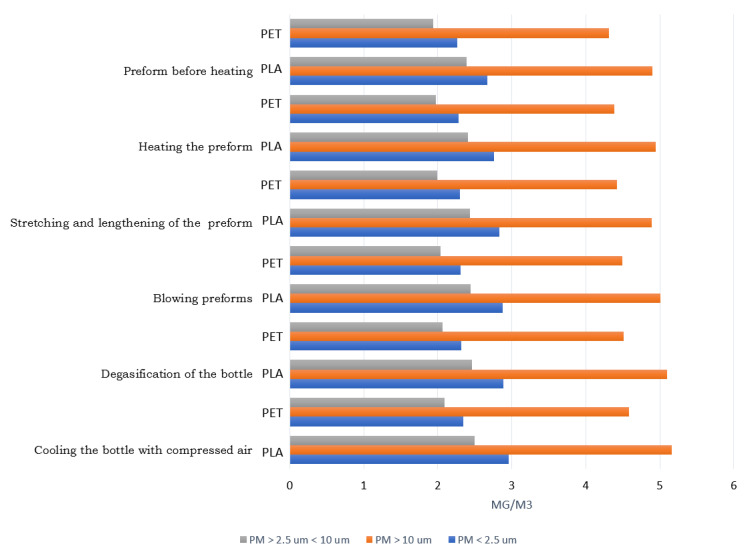
Dust concentrations PM < 2.5, PM > 10, 2.5 < PM < 10 in the process of shaping for PET and PLA bottles.

Bearing in mind the negative impact of airborne dust, special attention was paid to fine dust fractions and their impact on human health. To date, no threshold has been set below which PM10 and PM2.5 particulate matter would not pose a risk. The analyses show that the highest concentration levels in the entire PET bottle shaping process were determined for PM10 particles, while the lowest concentration levels were determined for coarse particles in the PM2.5–10 range (Figure 15).

In the case of the process of shaping PLA bottles, lower concentration values of all the technological operations were noted. Both fine and coarse particles were characterized by lower concentration parameters, which shows that the process of shaping biodegradable bottles is more environmentally friendly.

## 4. Discussion

The main goal of the study was achieved by conducting an ecological analysis of the life cycle of the beverage bottle production process. The analyses were based on the LCA method, and CML 2 and ReCiPe2016 were used as calculation procedures. The results were summarized separately for the two types of single-use bottles (PET bottle and PLA bottle).

In conjunction with the greater responsibility of producers, modern recycling devices, and announcements of changes in regulations, the amount of plastic will be reduced. It is estimated that even 8 million tons of plastic end up in the seas and oceans in the world each year. This effect can be avoided if more and more biodegradable packaging becomes available on the market. According to data from the European Commission, 25 million tons of plastic waste is produced in Europe every year, but only one in three is recycled [37]. Manufacturers of plastic packaging are increasingly looking for innovative, pro-ecological solutions that will somehow contribute to reducing the negative impact on the environment.

Comparing the impact categories, the results were presented in accordance with the impact category units adopted by LCA. Based on the conducted analysis, it can be concluded that the PET bottle production process showed a higher level of harmful environmental impact than the PLA bottle production process. The greatest number of potential negative impacts on the environment, in the case of all the studied objects, was recorded for the impact category: processes related to marine aquatic ecotoxicity (from 89.36 to 51.53 kg 1,4-DB eq for PLA bottle and from 91.65 to 57.99 kg 1,4-DB eq for PET bottle). Another category negatively affecting the condition of the natural environment is human toxicity, for which higher emission values were noted during the cooling process of the finished PET bottle (0.085 kg 1,4-DB eq). In turn, in the case of the freshwater aquatic ecotox category, higher levels of substance emissions were reported for the cooling process of the PLA bottle (0.049 kg 1,4-DB eq). In addition, notable differences in the results were observed for the global warming category, where during the first operating process—preform before heating—the PET bottle production process showed the greatest negative environmental impact (Figure 10).

The results show that in most cases, the polymer materials used today contribute to the most efficient use of resources, taking into account the energy balance [38]. Polymers based on renewable resources are no better than conventional plastics based on fossil fuels because of their origin. Plastics sourced from renewable resources can contribute to reducing greenhouse gas emissions in the future if the right raw materials are chosen, and further appropriate waste management methods are chosen [39]. Similar research results have been published in [15,16,17], in which, in most of the impact categories, raw materials of natural origin generally give better environmental performance than raw materials used for the production of PET bottles.

The analysis evaluation of the effectiveness of the material forming process PLA and PET bottles for beverages proposes a number of measures to improve and corrective measures.

When analyzing the research results, it is recommended to focus the measures taken in the field of water management by improving the efficiency of water consumption and reusing it in the bottling process and in the bottle shaping process. In addition, it is proposed to implement a management system covering wastewater generated in the production process and full use of rainwater to reduce and prevent pollution.

Conscious management of post-consumer packaging should focus on reducing the amount of waste and improving the environment.

The implementation of LCA may bring tangible benefits in the environmental assessment of production processes responsible for the production of packaging [40]. This means that in addition to the environmental effects of the bottle production process, the LCA analysis also takes into account the environmental impact of all materials and energy used (including the use of PET and PLA preforms). The use of LCA analysis allows for a comprehensive inventory of input and output data in the life cycle of the bottle production process. It also allows for an environmental assessment both in individual impact categories, such as greenhouse gas emissions and fossil fuel depletion. This permits a comparative analysis of the different processes of production of bottles made from non-renewable resources and biodegradable material in the landfill. The use of LCA can be used to support environmental management systems and also as a supplementary element for the environmental impact assessment. LCA can also be used as a decision-making tool related to the environmental impact of manufacturing activities. Environmental indicators obtained through the use of LCA can be used to support decisions made in the organization related to the analysis and assessment of the environmental aspects of the operation of production plants in Poland. LCA may also be an element of a strategic, multi-faceted analysis of the effectiveness of Polish industry [41,42].

## Figures and Tables

**Table 1:** The results of Life Cycle Inventory for the production of 1 bottle 1000 mL made with PLA [26].

Technological Operations	Ecoinvent Activity	Amount PLA Bottle
Raw material acquisition		
PLA preform mass	Polylactide, granulate {GLO} market for/Alloc Def, S	18.24 g
Electrical energy	Electricity, medium voltage {PL} market for/Alloc Def, S	0.368 kWh
Preform heating		
Electrical energy (infrared lamp 100 kW)	Electricity, medium voltage {PL} market for/Alloc Def, S	3.2 kWh
Electrical energy (infrared lamps 200 kW)		6.4 kWh
Electrical energy (supply chain)		0.16 kWh
Preform stretching and extending		
Electrical energy	Electricity, medium voltage {PL} market for/Alloc Def, S	6.95 kWh
Compressed air	Compressed air, 1000 kPa gauge {RER} compressed air production/Alloc Def U	0.0016 kg/m^3^
Preform pressure shaping		
Electrical energy	Electricity, medium voltage {PL} market for/Alloc Def, S	5.66 kWh
Bottle degasifying		
Electrical energy	Electricity, medium voltage {PL} market for/Alloc Def, S	1.01 kWh
Bottle cooling		
Electrical energy	Electricity, medium voltage {PL} market for/Alloc Def, S	0.71 kWh
Water in a closed circulation	Tap water {Europe without Switzerland} market for/Alloc Def, S	2.4 m^3^

**Table 2:** The total impact of the production of bottles on the environment.

Impact Category	Unit	PET Bottle	PLA Bottle
Abiotic depletion	kg Sb eq	8.18	4.89
Acidification	kg SO_2_ eq	9.90 × 10^−6^	0.00004
Eutrophication	kg PO_4_ eq	1.47	1.47
Global warming (GWP100)	kg CO_2_ eq	6.57	38.14
Ozone layer depletion (ODP)	kg CFC-11 eq	3.36 × 10^−5^	0.00004
Human toxicity	kg 1,4-DB eq	485.12	354.27
Fresh water aquatic ecotox.	kg 1,4-DB eq	223.24	258.36
Marine aquatic ecotoxicity	kg 1,4-DB eq	481,609.65	456,003.83
Terrestrial ecotoxicity	kg 1,4-DB eq	1.99	4.08
Photochemical oxidation	kg C_2_H_4_ eq	0.19	0.17

## Data Availability

The data presented in this study are available on request from the corresponding author.

## References

[1] Lloyd S.M., Ries R. (2007). Characterizing, Propagating, and Analyzing Uncertainty in Life-Cycle Assessment: A Survey of Quantitative Approaches. J. Ind. Ecol..

[2] Piasecka I., Baldowska-Witos P., Piotrowska K., Tomporowski A. (2020). Eco-Energetical Life Cycle Assessment of Materials and Components of Photovoltaic Power Plant. Energies.

[3] Maurice B., Frischknecht R., Coelho-Schwirtz V., Hungerbühler K. (2000). Uncertainty analysis in life cycle inventory. Application to the production of electricity with French coal power plants. J. Clean. Prod..

[4] Wang E., Shen Z., Neal J., Shi J., Berryman C., Schwer A. (2012). An AHP-weighted aggregated data quality indicator (AWADQI) approach for estimating embodied energy of building materials. Int. J. Life Cycle Assess.

[5] (2013). Act of June 13, 2013 on the management of packaging and packaging waste. J. Laws.

[6] Frischknecht R., Jungbluth N., Althaus H.-J., Doka G., Dones R., Heck T., Hellweg S., Hischier R., Nemecek T., Rebitzer G. (2005). The ecoinvent Database: Overview and Methodological Framework (7 pp). Int. J. Life Cycle Assess..

[7] Weidema B.P., Wesnæs M.S. (1996). Data quality management for life cycle inventories—an example of using data quality indicators. J. Clean. Prod..

[8] Sonnemann G.W., Schuhmacher M., Castells F. (2003). Uncertainty assessment by a Monte Carlo simulation in a life cycle inventory of electricity produced by a waste incinerator. J. Clean. Prod..

[9] (2003). International Organization for Standarization ISO/TR 14047:2003-Environmental Management—Life Cycle Impact Assessment—Examples of Application of ISO 14042 2003.

[10] (2002). International Organization for Standarization ISO/TS 14048:2002-Environmental Management—Life Cycle Assessment—Data Documentation Format 2002.

[11] (2006). International Organization for Standardization ISO 14040:2006-Environmental Management—Life Cycle Assessment—Principles and Framework 2006.

[12] (2006). International Organization for Standardization ISO-ISO 14044:2006-Environmental Management—Life Cycle Assessment—Requirements and Guidelines 2006.

[13] Shen L., Worrell E., Patel M.K. (2010). Open-loop recycling: A LCA case study of PET bottle-to-fibre recycling. Resour. Conserv. Recycl..

[14] Bałdowska-Witos P., Kruszelnicka W., Kasner R., Rudnicki J., Tomporowski A., Flizikowski J. (2019). Impact of the plastic bottle production on the natural environment. Part 1. Application of the ReCiPe 2016 assessment method to identify environmental problems. Przemysl. Chem..

[15] Walkerab S., Rothmana R. (2020). Life cycle assessment of bio-based and fossil-based plastic: A review. J. Cleaner Prod..

[16] Horowitz N., Frago J., Dongyan M. (2018). Life cycle assessment of bottled water: A case study of Green2O products. Waste Manag..

[17] Chen L., Pelton R.E.O., Smith T.M. (2016). Comparative life cycle assessment of fossil and bio-based polyethylene terephthalate (PET) bottles. J. Clean. Prod..

[18] Kowalski Z., Kulczycka J. (2005). Ocena cyklu życia LCA jako podstawowy czynnik oceny czystszych produkcji. Odzysk odpadów–technologie i możliwości. Proceedings of the Materiały Konfrerencji Waste Recycling.

[19] Benetto E., Dujet C., Rousseaux P. (2008). Integrating fuzzy multicriteria analysis and uncertainty evaluation in life cycle assessment. Environ. Model. Softw..

[20] Egilmez G., Gumus S., Kucukvar M., Tatari O. (2016). A fuzzy data envelopment analysis framework for dealing with uncertainty impacts of input–output life cycle assessment models on eco-efficiency assessment. J. Clean. Prod..

[21] Lasvaux S., Schiopu N., Habert G., Chevalier J., Peuportier B. (2014). Influence of simplification of life cycle inventories on the accuracy of impact assessment: Application to construction products. J. Clean. Prod..

[22] Chou J.-S., Yeh K.-C. (2015). Life cycle carbon dioxide emissions simulation and environmental cost analysis for building construction. J. Clean. Prod..

[23] Geisler G., Hellweg S., Hungerbühler K. (2005). Uncertainty Analysis in Life Cycle Assessment (LCA): Case Study on Plant-Protection Products and Implications for Decision Making (9 pp + 3 pp). Int. J. Life Cycle Assess.

[24] Chevalier J.-L., Téno J.-F.L. (1996). Life cycle analysis with ill-defined data and its application to building products. Int. J. Life Cycle Assess..

[25] Gaňa D., Liptáková T., Markovičová L. (2019). Comparison of the properties of the original and applied LDPE foils in returned bottles. Prod. Eng. Arch..

[26] Bałdowska-Witos P., Kruszelnicka W., Kasner R., Tomporowski A., Flizikowski J., Kłos Z., Piotrowska K., Markowska K. (2020). Application of LCA Method for Assessment of Environmental Impacts of a Polylactide (PLA) Bottle Shaping. Polymers.

[27] Mielczarek K., Krynke M. (2018). Plastic production machinery–the evaluation of effectiveness. Prod. Eng. Arch..

[28] Reap J., Roman F., Duncan S., Bras B. (2008). A survey of unresolved problems in life cycle assessment. Int. J. Life Cycle Assess..

[29] Miller S.A., Moysey S., Sharp B., Alfaro J. (2013). A Stochastic Approach to Model Dynamic Systems in Life Cycle Assessment. J. Ind. Ecol..

[30] Thiel C.L., Campion N., Landis A.E., Jones A.K., Schaefer L.A., Bilec M.M. (2013). A Materials Life Cycle Assessment of a Net-Zero Energy Building. Energies.

[31] Peters G.P. (2006). Efficient algorithms for Life Cycle Assessment, Input-Output Analysis, and Monte-Carlo Analysis. Int. J. Life Cycle Assess..

[32] Heijungs R., Tan R.R. (2010). Rigorous proof of fuzzy error propagation with matrix-based LCI. Int. J. Life Cycle Assess.

[33] Imbeault-Tétreault H., Jolliet O., Deschênes L., Rosenbaum R.K. (2013). Analytical Propagation of Uncertainty in Life Cycle Assessment Using Matrix Formulation. J. Ind. Ecol..

[34] Ciroth A., Fleischer G., Steinbach J. (2004). Uncertainty calculation in life cycle assessments. Int. J. Life Cycle Assess..

[35] Piotrowska K., Kruszelnicka W., Bałdowska-Witos P., Kasner R., Rudnicki J., Tomporowski A., Flizikowski J., Opielak M. (2019). Assessment of the Environmental Impact of a Car Tire throughout Its Lifecycle Using the LCA Method. Materials.

[36] Bałdowska-Witos P., Kruszelnicka W., Kasner R., Tomporowski A., Flizikowski J., Mrozinski A. (2019). Impact of the plastic bottle production on the natural environment. Part 2. Analysis of data uncertainty in the assessment of the life cycle of plastic beverage bottles using the Monte Carlo technique. Przem. Chem..

[37] (2018). Report European Commission: European Strategy for Plastics in a Circular Economy. https://ec.europa.eu/info/research-and-innovation/research-area/environment/circular-economy/plastics-circular-economy_en.

[38] Edelen A., Ingwersen W. (2016). Guidance on Data Quality Assessment for Life Cycle Inventory Data.

[39] Abdeev B.M., Azamatkyzy S.A., Cyganiuk J., Doudkin M.V., Idzikowski A., Przystupa F.W., Pustałka A., Sakimov M.A., Spangemacher L. (2017). Modeling of Machinery Processes.

[40] Cyganiuk J., Doudkin M.V., Frohilich S.R., Idzikowski A., Kim A.I., Kruszelnicka W., Sokolski P., Schuning T., Tomporowski A., Vaviolov A.V. (2017). Modeling of Machinery Processes.

[41] Mannheim V. (2011). Empirical and scale-up modeling in stirred ball mills. Chem. Eng. Res. Des..

[42] Mannheim V., Fehér Z., Siménfalvi Z., Tóthné Szita K., Jármai K., Voith K. (2019). Innovative solutions for the building industry to improve sustainability performance with Life Cycle Assessment modelling. Book Solutions for Sustainable Development.

